# Screening potential FDA-approved inhibitors of the SARS-CoV-2 major protease 3CL^pro^ through high-throughput virtual screening and molecular dynamics simulation

**DOI:** 10.18632/aging.202703

**Published:** 2021-03-07

**Authors:** Wen-Shan Liu, Han-Gao Li, Chuan-Hua Ding, Hai-Xia Zhang, Rui-Rui Wang, Jia-Qiu Li

**Affiliations:** 1 Shandong Key Laboratory of Clinical Applied Pharmacology, Department of Pharmacy, Affiliated Hospital of Weifang Medical University, Weifang 261031, Shandong, China; 2 Department of Oncology, Affiliated Hospital of Weifang Medical University, Weifang 261031, Shandong, China; 3 Tianjin Key Laboratory on Technologies Enabling Development of Clinical Therapeutics and Diagnostics (Theranostics), School of Pharmacy, Tianjin Medical University, Tianjin 300070, China

**Keywords:** SARS-CoV-2, COVID-19, Indinavir, molecular docking, molecular dynamics simulation

## Abstract

It has been confirmed that the new coronavirus SARS-CoV-2 caused the global pandemic of coronavirus disease 2019 (COVID-19). Studies have found that 3-chymotrypsin-like protease (3CL^pro^) is an essential enzyme for virus replication, and could be used as a potential target to inhibit SARS-CoV-2. In this work, 3CL^pro^ was used as the target to complete the high-throughput virtual screening of the FDA-approved drugs, and Indinavir and other 10 drugs with high docking scores for 3CL^pro^ were obtained. Studies on the binding pattern of 3CL^pro^ and Indinavir found that Indinavir could form the stable hydrogen bond (H-bond) interactions with the catalytic dyad residues His41-Cys145. Binding free energy study found that Indinavir had high binding affinity with 3CL^pro^. Subsequently, molecular dynamics simulations were performed on the 3CL^pro^ and 3CL^pro^-Indinavir systems, respectively. The post-dynamic analyses showed that the conformational state of the 3CLpro-Indinavir system transformed significantly and the system tended to be more stable. Moreover, analyses of the residue interaction network (RIN) and H-bond occupancy revealed that the residue-residue interaction at the catalytic site of 3CL^pro^ was significantly enhanced after binding with Indinavir, which in turn inactivated the protein. In short, through this research, we hope to provide more valuable clues against COVID-19.

## INTRODUCTION

A severe acute respiratory syndrome coronavirus 2 (SARS-CoV-2) is the causative agent of coronavirus disease 2019 (COVID-19). The virus emerged in Wuhan, China at the end of December 2019 and quickly spread to the world [1, 2]. On March 11, 2020, the World Health Organization (WHO) declared the COVID-19 outbreak a pandemic. The virus is highly infectious. As of October 23, 2020, COVID-19 has infected more than 60 million people, caused more than 1.4 million deaths, and the number of patients and deaths continues to increase, posing a serious health threat to the world. The symptoms of COVID-19 infection include high fever, cough and shortness of breath, and severe cases can lead to kidney failure and death [3, 4]. Currently, there are no approved drugs or vaccines against COVID-19.

SARS-CoV-2 belongs to the beta coronavirus group [5]. Generally, β-coronaviruses produce an 800-kDa polypeptides during genome transcription. The polypeptide is proteolytically cleaved to produce various proteins [2, 6]. The proteolytic process is mediated by papain-like protease (PLpro) and 3-chymotrypsin-like protease (3CL^pro^). 3CL^pro^ cleaves polyprotein at 11 distinct sites to generate various non-structural proteins that are important for viral replication [7, 8]. In view of the key role of 3CL^pro^ in viral replication, 3CL^pro^ is used as a potential target against COVID-19 inhibitors.

**Figure 1:**
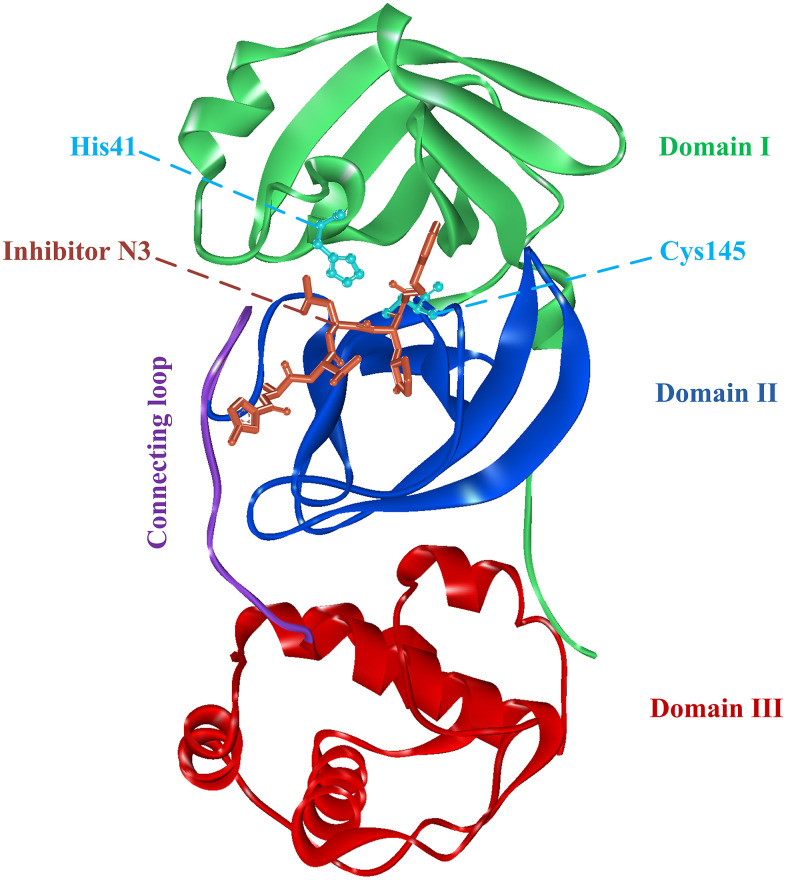
**The complex structure of 3CL^pro^ protein and synthetic peptidomimetic inhibitor N3.** 3CL^pro^ contains three domains, namely domain I, domain II, and domain III, and a connecting loop. Residues His41 and Cys145 are important catalytic dyads.

Since the outbreak of COVID-19, researchers had obtained multiple structures of SARS-CoV-2 protein using X-ray diffraction or cryo-electron microscopy. The structure used in this study was the complex crystal structures of SARS-CoV-2 3CL^pro^ protein combined with a synthetic peptidomimetic inhibitor N3 (PDB: 6LU7, Figure 1) [9]. The 3CL^pro^ protein has three important domains I-III, corresponding to residues Phe8-Tyr101, Lys102-Pro184, and Thr201-Val303. There is also a connecting loop corresponding to residues Phe185-Ile200, which connects domains II and III. The 3CL^pro^ protein has an important catalytic dyads consisting of His41 and Cys145, and the substrate-binding site is located in the cleft between domains I and II [9–11].

In this study, we used the SARS-COV-2 3CL^pro^ protein as the target to conduct high-throughput virtual screening of the FDA-approved drugs, and sorted them according to the level of docking scores to find potential inhibitors for effective treatment of SARS-COV-2. Taking the inhibitor Indinavir with the highest docking score as a representative, we further explored the binding pattern of 3CL^pro^ and Indinavir, and calculated the binding free energy between them to evaluate the binding affinity. Subsequently, in order to further investigate the effect of the Indinavir on the conformation of 3CL^pro^ protein, 100 ns molecular dynamics (MD) simulations were performed for the 3CL^pro^ system and the 3CL^pro^-Indinavir complex system, respectively, and post-dynamic analyses were performed. The stability of the two systems was evaluated by using root mean square deviation (RMSD) and root mean square fluctuation (RMSF). Principal component analysis (PCA) and dynamic cross-correlation diagram (DCCM) were used to explore the differences in the conformational state and correlated motions of the two systems. Through the analyses of the residue interaction network (RIN) and residue-residue hydrogen bond (H-bond) occupancy, the differences in the interaction between the two systems were explored, and the underlying reason for the conformational differences between the two systems were further revealed. Based on the above research, we hoped that this research would provide valuable information for the clinical treatment against COVID-19.

## RESULTS

### Result on high-throughput virtual screening

Virtual screening was an effective method for the development of new drugs with desired characteristics and structural diversity [12]. In this study, through the virtual screening method based on docking, high-throughput virtual screening was performed on the FDA-approved drugs from the ZINC database. Before docking screening, in order to evaluate the reliability of the CDOCKER model, the ligand N3 in the original crystal structure was reinserted into the binding pocket. It was found that the RMSD between the docking conformation of the original ligand and the eutectic conformation was 0.8674Å (less than 2Å), indicating that the docking method and parameters were reliable. After that, all compounds were docked to the active site of 3CL^pro^ protein and sorted according to the level of docking score. Through virtual screening, we had obtained a number of valuable drugs against COVID-19. Table 1 listed the top 10 drugs with the docking score. Among them, Indinavir, as an anti-HIV protease inhibitor, had the highest docking score with 3CL^pro^ protein.

**Table 1:** Top 10 FDA-approved drugs with docking score based on high-throughput virtual screening.

**ID**	**Name**	**Chemical structure**	**Docking score**
ZINC22448696	Indinavir	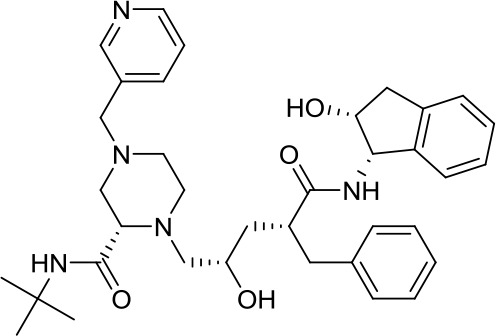	49.5328
ZINC3944422	Ritonavir	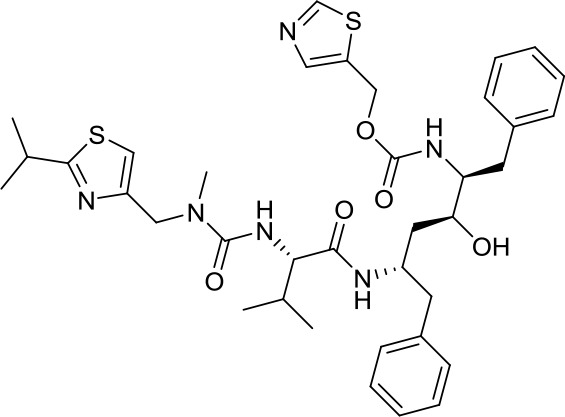	49.4166
ZINC3914596	Saquinavir	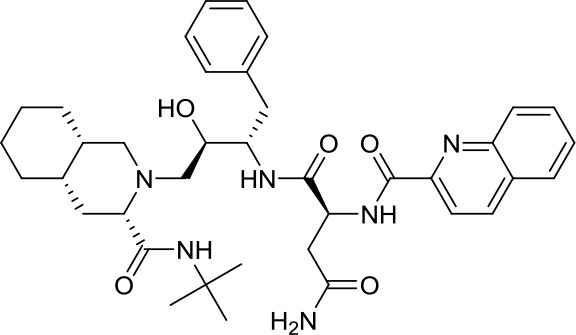	49.3129
ZINC3951740	Lopinavir	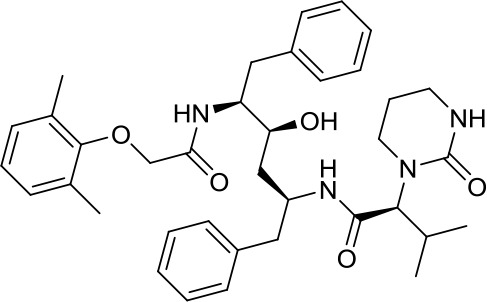	49.0524
ZINC85537014	Cobicistat	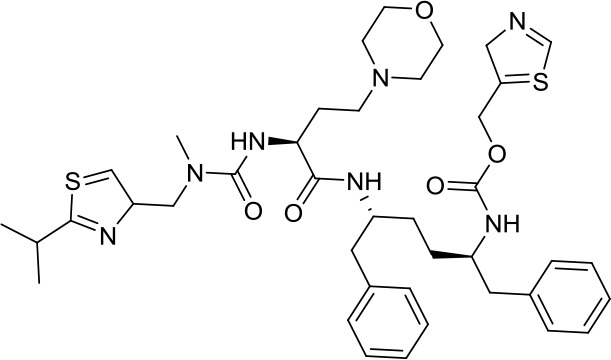	48.9127
ZINC100016058	Tipranavir	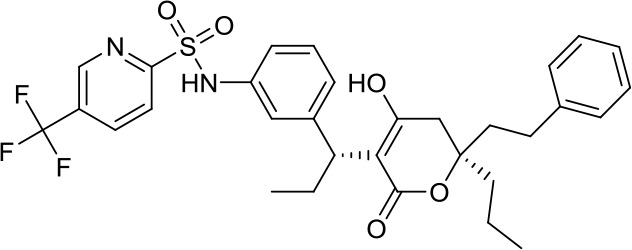	48.8735
ZINC3831151	Montelukast	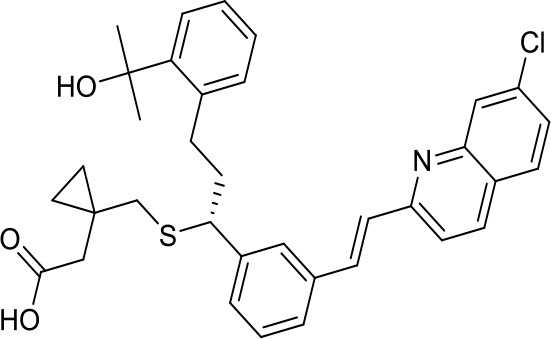	48.8146
ZINC49841054	Kyprolis	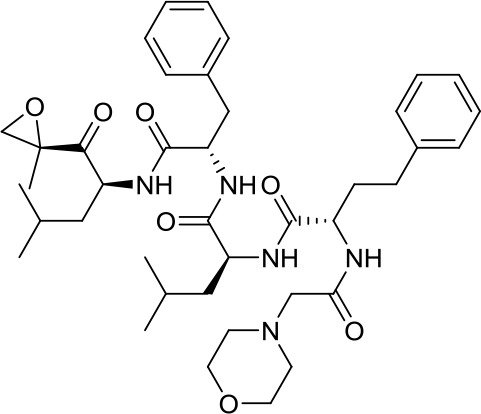	48.8093
ZINC29571072	Isavuconazonium	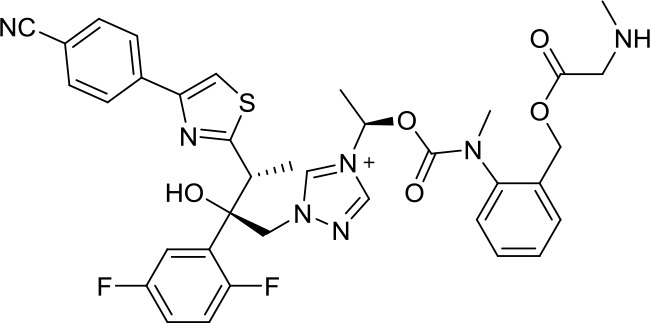	47.9368
ZINC14879972	Tigecycline	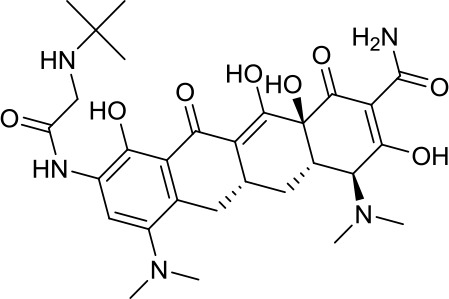	47.9302

### Investigation on the binding pattern

**Figure 2:**
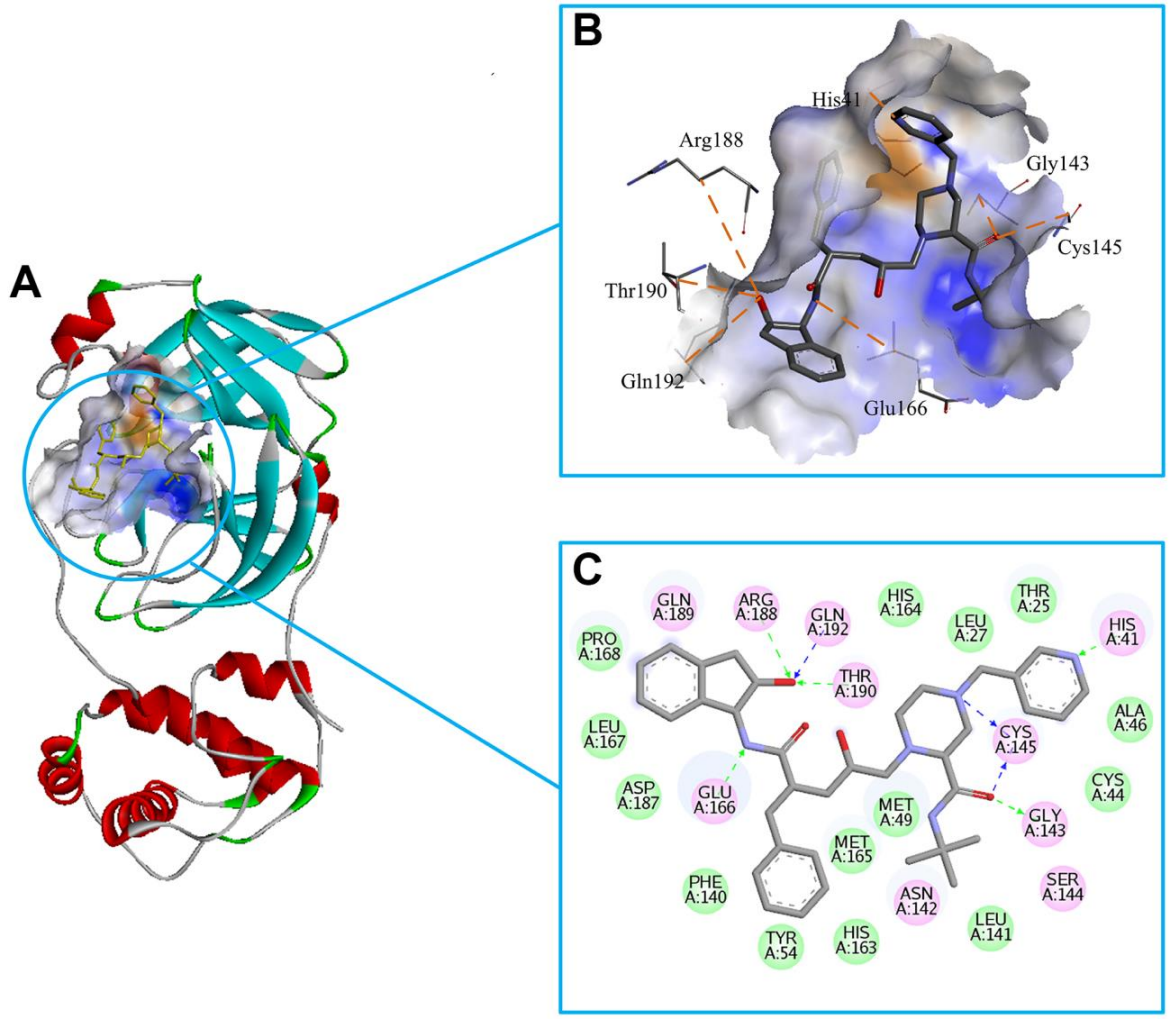
**Investigation on the binding pattern of 3CL^pro^ and Indinavir.** (**A**) The location of the binding pocket between 3CL^pro^ and Indinavir. (**B**) The enlarged view of the binding pocket of 3CL^pro^ and Indinavir. (**C**) The interaction 2D diagram between 3CL^pro^ and Indinavir. The green rectangles represent VDW interaction. The pink rectangles represent charge and H-bond interactions. Here, the H-bond interactions with the main residues are indicated by the green dashed arrow pointing to the electron donor, and the H-bond interactions with the side chain residues are indicated by the blue dashed arrow pointing to the electron donor.

We chose the Indinavir to explore its binding pattern with 3CL^pro^ protein. Figure 2A showed that Indinavir was docked to the binding site of the 3CL^pro^ protein, Figure 2B showed the binding pocket of the Indinavir and protein, and Figure 2C showed a 2D diagram of the interaction between the Indinavir and protein. It could be seen from the 2D diagram that the Indinavir could form 7 stable H-bond interactions with residues His41, Gly143, Cys145, Glu166, Arg188, Thr190 and Gln192, and formed van der waals (VDW) interactions with residues Thr25, Leu27, Cys44, Ala46, Met49, Tyr54, Phe140, Leu141, His163, His164, Met165, Leu167, Pro168 and Asp187. It was worth noting that the conserved catalytic dyad residues His41 and Cys145 in the protein were tightly bound with the Indinavir, which was beneficial to stabilize the protein. Based on the multiple stable interactions formed between the protein and the ligand, the Indinavir could play a role in inhibiting the activity of 3CL^pro^ protein.

### The calculation of binding free energy

To better explore how ligand affected the stability and interaction of protein-ligand complexes, the binding free energies of protein-ligand complexes were calculated using the MM/PBSA algorithm. binding free energy was described by four characteristic terms, including VDW interaction energy, electrostatic energy, polar solvation free energy and non-polar solvation free energy. VDW, electrostatic interaction and nonpolar solvation energy had negative contribution to the total interaction energy, while only polar solvation energy had a positive contribution to the total free binding energy, which meant that VDW, electrostatic interaction and non-polar solvation energy were beneficial to enhance the stability of the complex systems. According to Table 2, it could be seen that the binding free energy between 3CL^pro^ protein and Indinavir was -239.796 kJ/mol, indicating that they had high binding affinity.

**Table 2:** The binding free energy of 3CL^pro^ and Indinavir.

**Complex**	**Van der Waal (kJ/mol)**	**Electrostatic (kJ/mol)**	**Polar solvation (kJ/mol)**	**Non-polar solvation (kJ/mol)**	**Binding energy (kJ/mol)**
3CL^pro^-Indinavir	-128.153	-173.386	89.208	-27.465	-239.796

**Figure 3:**
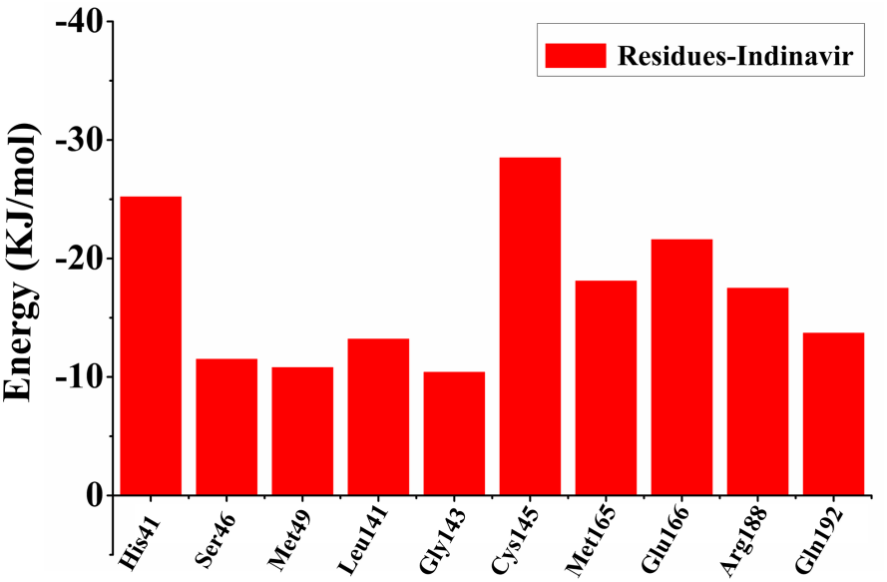
**The first 10 main residues in 3CL^pro^ that contribute to the binding free energy of 3CL^pro^ and Indinavir.** The ordinate indicates the value of binding free energy, and the abscissa indicates the residue.

In order to find out the contribution of different residues to the interaction between the protein-ligand complex, the free energy decomposition method was used to decompose the interaction energy into single residue, and further explored the differences in the sources of binding affinity between Indinavir and 3CL^pro^ protein. The result of free energy decomposition was shown in Figure 3. Among them, the first 10 main residues in the 3CL^pro^ protein that had higher binding free energies with the Indinavir were His41, Ser46, Met49, Leu141, Gly143, Cys145, Met165, Glu166, Arg188 and Gln192, respectively. These main residues were mainly distributed in regions I and II, which could help enhance the binding affinity between the 3CL^pro^ protein and Indinavir.

### Research on stability and flexibility of simulation system

**Figure 4:**
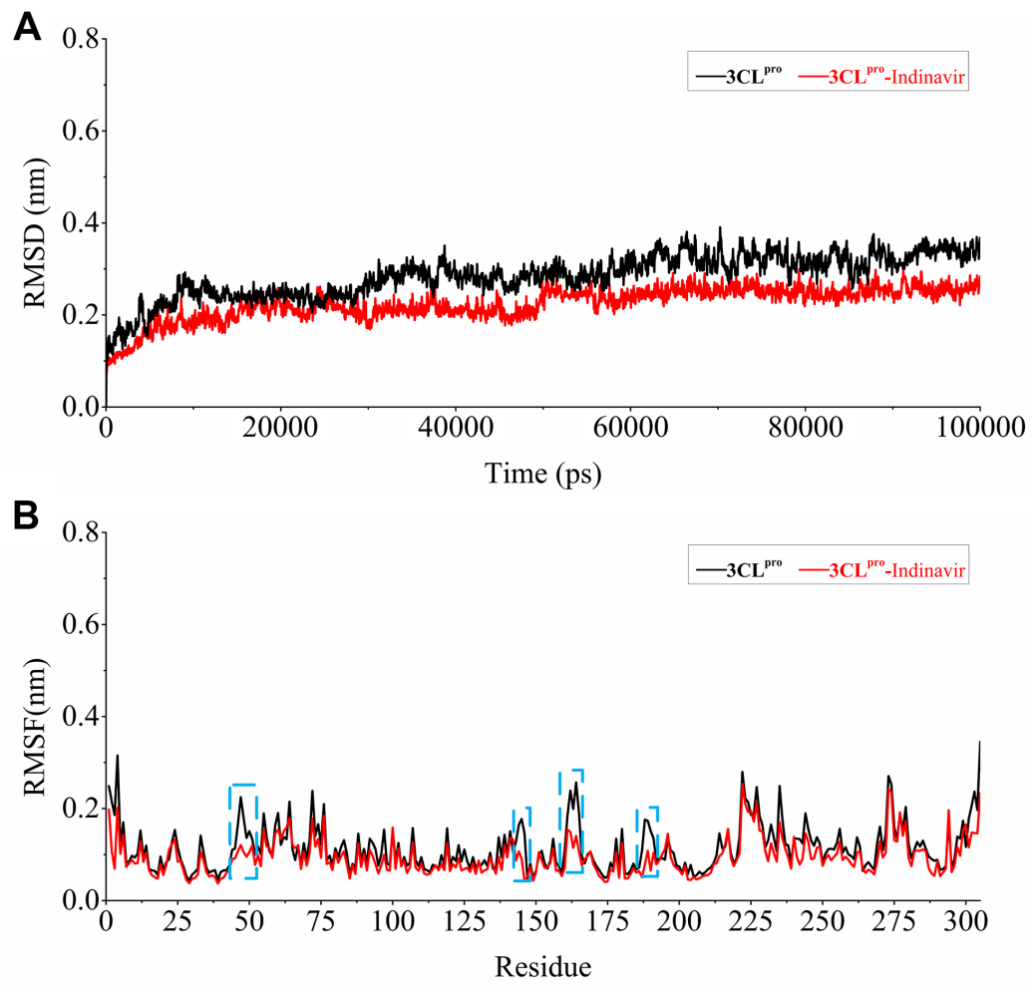
**Stability evaluation of 3CL^pro^ system and 3CL^pro^-Indinavir system.** (**A**) RMSD of all main chain atoms of 3CL^pro^ system and 3CL^pro^-Indinavir system. (**B**) RMSF of side chain atoms of 3CL^pro^ system and 3CL^pro^-Indinavir system. The black line indicates the result of the 3CL^pro^ system, and the red line indicates the result of the 3CL^pro^-Indinavir system. In addition, these regions (residues Glu47-Tyr54, Phe140-Cys145, His163-Pro168 and Val186-Thr190) are highlighted with blue dotted box.

In order to explore the relevant information of protein systems over time, 100 ns molecular dynamics simulations of 3CL^pro^ system (without ligand) and 3CL^pro^-Indinavir complex system were carried out. The RMSD was used to evaluate the difference of the protein main chain from its initial structural conformation to its final position. Through RMSD analysis, the conformational stability of the system during MD simulation could be evaluated. The fluctuation amplitude of the RMSD curve was inversely related to the stability of the protein, that was, the smaller the fluctuation, the more stable the system. Figure 4A showed the RMSD curves of the skeleton atoms in the 3Cl^pro^ system (black curve) and 3CL^pro^-Indinavir complex system (blue curve). It could be found from the Figure 3 that these two systems tended to converge around 5 ns and reach equilibrium afterwards. The average RMSD values in the 3CL^pro^ and 3CL^pro^-Indinavir systems were 0.2712 nm and 0.2205 nm, respectively. Since the simulation trajectories of the two systems were stable within 5-100 ns, the simulation trajectories of the last 95 ns were intercepted for later analysis.

Residues were protein components that determined its conformational characteristics. This ligand-residue interaction at the active site might induce conformational changes in protein structure and change its function. More specifically, the conformational change was the result of ligand-induced movement during ligand binding. Understanding ligand-induced conformational changes in protein structure was essential for rational drug design based on structure. The RMSF was a measure of the average atomic mobility of skeleton atoms (N, Cα, and C) during MD simulations and was used to assess the flexibility of the side chains of residues [13]. In order to explore the impact of each residue on protein flexibility, the RMSF fluctuation value was calculated, as shown in Figure 4B. The overall RMSF of the 3CL^pro^ and 3CL^pro^-Indinavir systems showed similar fluctuation trends, and the average values of the RMSF curves were about 0.1382 nm and 0.1266 nm, respectively. It was worth noting that in these regions (residues Glu47-Tyr54, Phe140-Cys145, His163-Pro168 and Val186-Thr190, marked with blue dotted boxes), compared with the 3CL^pro^ system, the 3CL^pro^-Indinavir system showed significantly reduced fluctuations. Moreover, since these four regions were mainly located in the catalytic binding region between protein domains I and II, this indicated that the 3CL^pro^ protein could exhibit lower flexibility and contribute to stabilize the protein conformation after binging with Indinavir.

### Investigation on the conformational transitions and dynamic domain motions of the 3CL^pro^ system and the 3CL^pro^-Indinavir complex system

**Figure 5:**
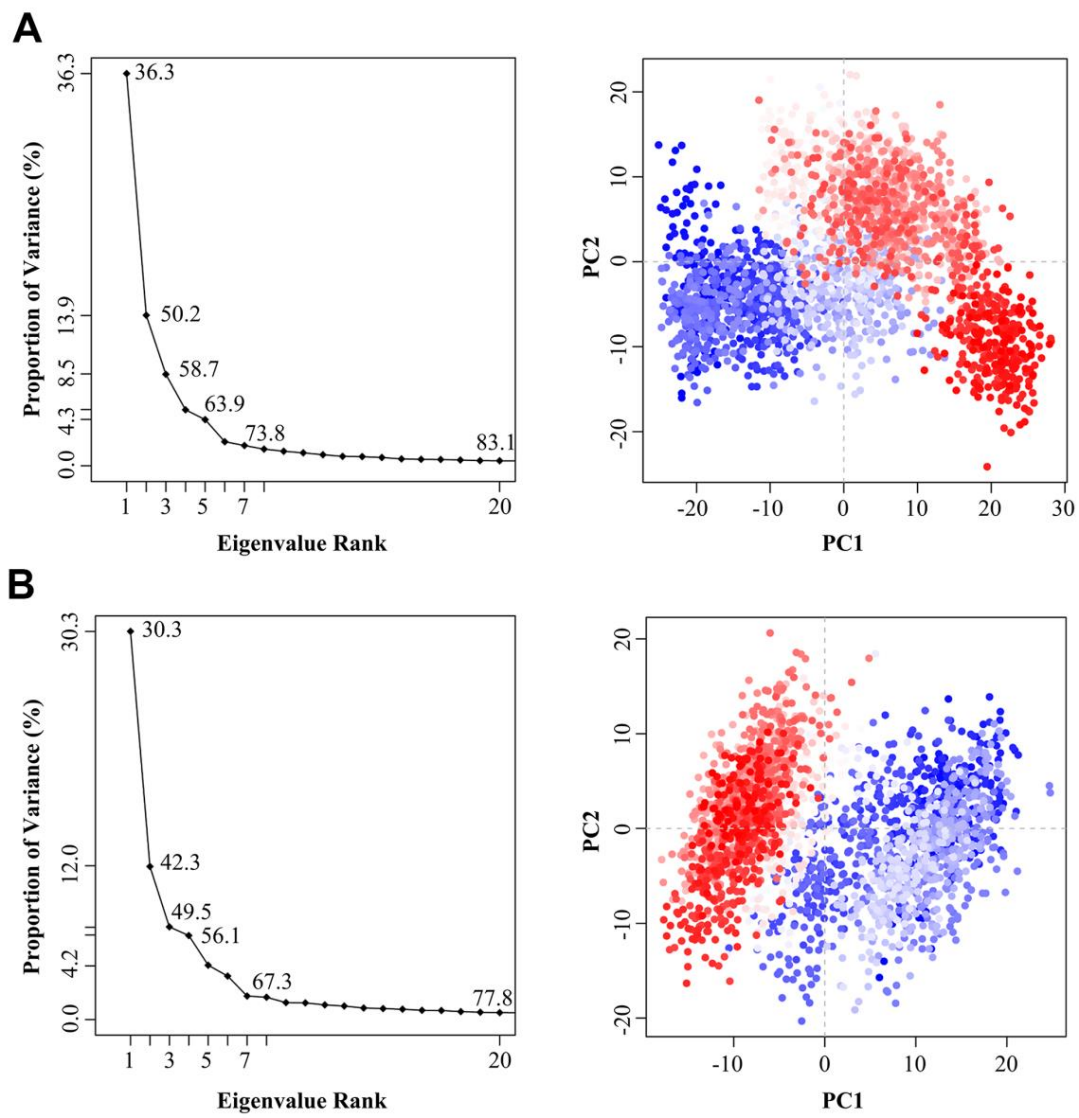
The variance contribution of the principal components and the PCA scatter plots generated along the projection of the first two eigenvectors (PC1 and PC2) in the space of the 3CL^pro^ (**A**) and 3CL^pro^-Indinavir (**B**) systems, respectively.

In order to explore the effect of the Indinavir on the conformational state of 3CL^pro^ protein, the last 95 ns of MD simulation trajectories were used to perform PCA on the Cα atoms from the 3CL^pro^ and 3CL^pro^-Indinavir systems. By projecting the simulated trajectories of the protein systems into the two-dimensional subspace spanned by the first three eigenvectors (PC1, PC2 and PC3), the PCA scatter plots of the 3CL^pro^ and 3Cl^pro^-Indinavir systems were obtained to explore the conformational transformation of the systems. As shown in Figure 5, the top 20 PCs of the 3CL^pro^ system and 3CL^pro^-Indinavir system accounted for 83.1% and 77.8% of the total change, respectively, indicating that compared with the 3CL^pro^ system, the 3CL^pro^-Indinavir system had a smaller phase space and a lower performance flexibility. In the 3CL^pro^ system, PC1 and PC2 contributed 36.3% and 13.9% to the variance, respectively, while other PCs contributed no more than 9.0%. In the 3CL^pro^-Indinavir system, PC1 and PC2 contributed 30.3% and 12.0% to the variance, while other PCs also contributed no more than 9.0%. This showed that the first two eigenvectors (PC1 and PC2) could largely reflect the transformation of the conformational state to a large extent, so we projected along the direction of the first two main components (PC1 and PC2) to generate PCA scatter plots (Figure 5). As shown in the figure, the PCA scatter diagram showed the conformational state of the two systems in the subspace, where the red dot represented the stable conformational state, the blue dot represented the unstable conformational state, and the intermediate state between the two conformations was represented by the white dots. The protein system periodically jumped between the two conformational states (blue and red). Comparing the scatter plots of the two systems, it could be seen that the conformational states of the protein after binding with the Indinavir had changed significantly. Compared with the irregular distribution of red and blue dots in the 3CL^pro^ system, the red and blue dots in the 3CL^pro^-Indinavir complex system were evenly distributed along the diagonal on both sides of the diagonal, indicating that the Indinavir contributed to stabilize the conformational state of 3CL^pro^ protein, which was consistent with the RMSF result.

**Figure 6:**
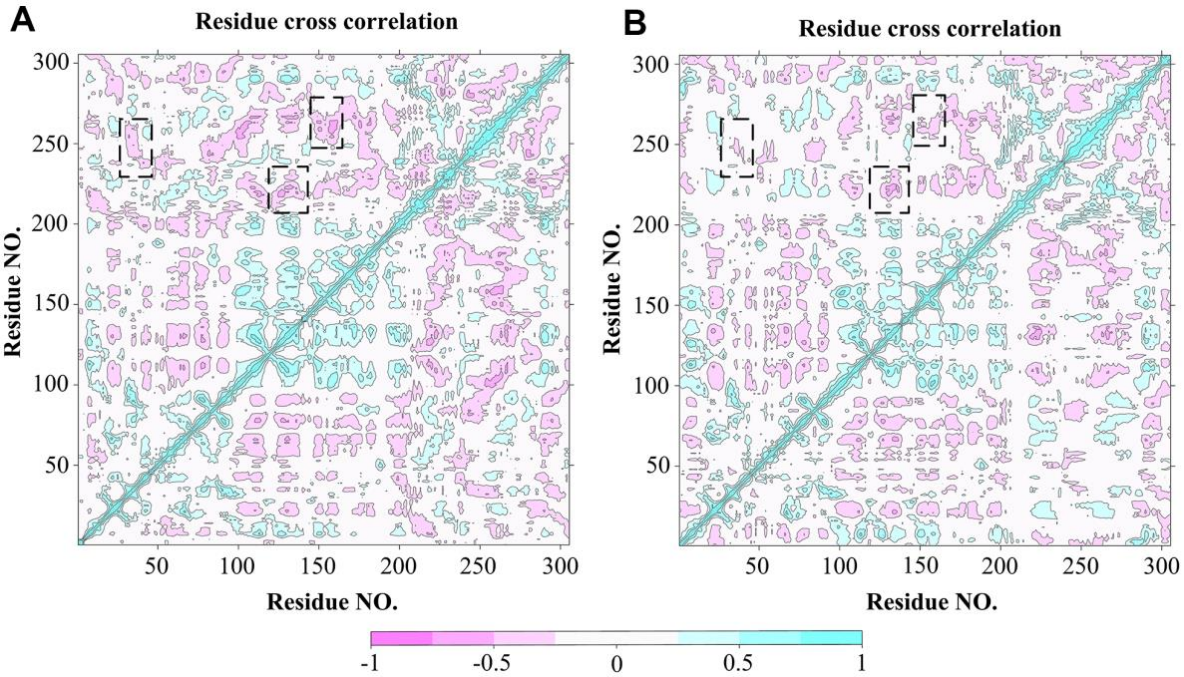
DCCM analysis of Cα atoms for 3CL^pro^ system (**A**) and 3CL^pro^-Indinavir system (**B**), respectively. The red regions indicate negative correlation, and the blue regions indicate positive correlation. The darker the color, the stronger the correlation. Regions with significant differences in correlated motions have been marked with black dashed boxes.

In order to explore the effect of the Indinavir on the conformational motions of 3CL^pro^ protein, DCCM analyses were performed on all Cα atoms in the 3CL^pro^ system and the 3CL^pro^-Indinavir complex system by extracting the last 95 ns simulated trajectories. The 2D diagrams of DCCM showed the correlated motions between residues during the whole simulation process (Figure 6). DCCM showed the overall correlation, which ranged from -1.0 to 1.0 (from dark purple to dark blue). Different colors were used to define different degrees of correlation between residues, and the darker the color, the stronger the correlation. The positive correlation (from 0 to 1) meant that the residues moved in the same direction, while the negative correlation (from -1 to 0) meant that the residues moved in the opposite direction. Comparing the DCCM diagrams of the two systems, it could be found that the correlated motions of the two systems were significantly different. Compared with the 3CL^pro^ system, the positive correlation motions of the entire 3CL^pro^-Indinavir system had not change much, but the negative correlation motions were significantly reduced. Especially in some important areas (marked with black dashed boxes), the correlated motions of the protein after binding with ligand would change significantly. Compared with the 3CL^pro^ system, in the 3CL^pro^-Indinavir system, the region (residues Leu250-Glu270) and region (residues Asn142-Met162), region (residues Gly215-Met235) and region (residues Gly120-Cys145), and region (residues Phe230-Cys265) and region (residues Leu30-Leu50) had significantly reduced negative correlation motions, which could lead to reduced flexibility of residues. Therefore, the reduction of negative correlation motions might be conducive to stabilizing the conformational state of protein domains I and II, so that the catalytically active site of the protein tended to be conserved, thereby inactivating the protein.

### The investigation of differences in the residue interactions between 3CL^pro^ system and 3CL^pro^-Indinavir system

**Figure 7:**
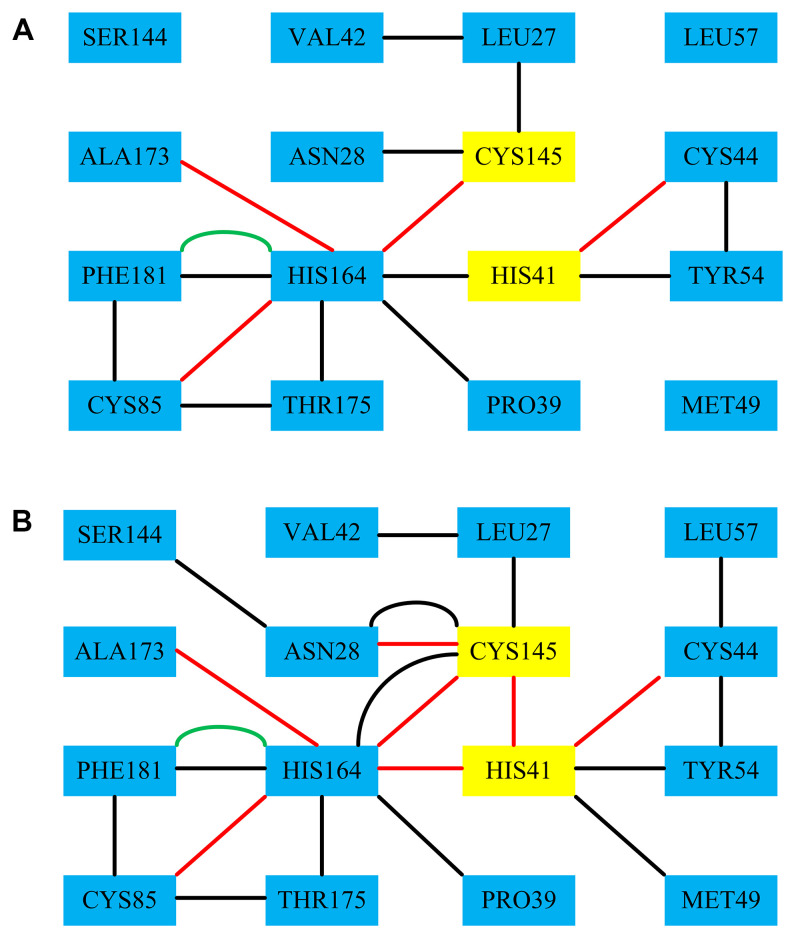
**The RINs results of the residues-residues around the catalytic dyads His41-Cys145 of the 3CL^pro^ system and the 3CL^pro^-Indinavir system, respectively.** Among them, the red line represents the H-bond interaction, the black line represents the VDW interaction, and the green line represents the Pi-Pi interaction.

In order to further explore the structure and function of 3CL^pro^ protein after binding with the Indinavir, RINs were performed on the 3CL^pro^ system and 3CL^pro^-Indinavir system to analyze the difference in the interaction between key residues. We focused on observing the differences in the residue-residue interactions around the catalytic dyads His41-Cys145 between the two systems. Figure 7 showed the RIN diagrams of the residue-residue interactions around the catalytic dyads His41-Cys145 of the two systems. According to the RIN results, the residue-residue interactions in the 3CL^pro^ protein would change significantly after binding with the Indinavir, which was mainly manifested in the significant increase in the H-bond interactions and VDW interactions formed between residues and residues. In the 3CL^pro^ system, Residue His41 formed an H-bond interaction with Cys44, and formed the VDW interactions with Tyr54 and His164. Residue Cys145 formed H-bond interaction with His164, and formed the VDW interactions with Leu27 and Asn28. In the 3CL^pro^-Indinavir system, residue His41 formed H-bond interactions with Cys44, Cys145 and His164, and formed VDW interactions with Met49 and Tyr54. Residue 145 formed the VDW interaction with Leu27, and formed H-bond interactions and VDW interactions with Asn28 and His164. It was worth noting that in the 3CL^pro^-Indinavir system, residue Cys145 formed new H bond interactions with Asn28 and His41, and formed new VDW interactions with His164. Residue His41 formed a new VDW interaction with Met49, and the interaction with His164 was transformed from VDW interaction to H-bond interaction. It could be clearly found that the interactions between the catalytic dyads His41-Cys145 and its surrounding residues had increased significantly. Therefore, the combination of 3CL^pro^ and Indinavir could significantly increase the residue-residue interaction at the catalytic site, which was conducive to stabilizing the protein conformation, and further revealed the underlying reason for the inactivation of 3CL^pro^ protein after binding with the Indinavir.

### The analysis on the H-bond occupancy

The residue-residue H-bond interaction was considered to be the most important factor affecting the conformational state of the protein. In order to verify whether the H-bond interaction shown on RINs in the 3CL^pro^ system and 3CL^pro^-Indinavir complex system was formed stably during the MD simulations, the H-bond occupancy was calculated. When the H-bond occupancy exceeded 50%, it indicated that the H-bond could be formed stably [14]. Table 3 showed the calculation results of the H-bond occupancy in the 3CL^pro^ system and the 3CL^pro^-Indinavir system during the 100 ns simulation process. In the 3CL^pro^ system, His41-Cys44, Cys145-Cys164, His164 and Cys85 and His164-Ala173 could form stable H-bond interactions, and the values of H-bond occupancy were 82.56%, 84.31%, 80.46% and 75.88%, respectively. In the 3CL^pro^-Indinavir system, His41-Cys44, His41-Cys145, His41-His164, Cys145-Asn28, Cys145-Cys164, His164-Cys85, His164-Cys85 and His164-Ala173 could all form stable H-bond interactions, and values of their H-bond occupancy were 92.33%, 98.28%, 89.37%, 87.64%, 95.98%, 88.19% and 91.49%, respectively. It was worth noting that in the 3CL^pro^-Indinavir system, His41-Cys145, His41-His164 and Cys145-Asn28 formed new H-bond interactions, and values of their H-bond occupancy exceeded 50%, indicating that these H-bond interactions were very stable, which further supported the results of RINs.

**Table 3:** The occupancy of H-bond interactions during the simulations of 3CL^pro^ system and 3CL^pro^-Indinavir system, respectively.

**Interaction type**	**Residue 1**	**Residue 2**	**Occupancy (%)** **(3CL^pro^)**	**Occupancy (%)** **(3CL^pro^- Indinavir)**
H bond	His41	Cys44	82.56	92.33
H bond	His41	Cys145	11.24	98.28
H bond	His41	His164	20.66	89.37
H bond	Cys145	Asn28	21.02	87.64
H bond	Cys145	Cys164	84.31	95.98
H bond	His164	Cys85	80.46	88.19
H bond	His164	Ala173	75.88	91.49

## DISCUSSION

COVID-19 continues to ravage the world, bringing a heavy burden to people all over the world, so we are eager to find a solution. 3CL^pro^ protein had become an attractive target for the treatment of SARS-COV-2 due to its key role in the virus replication cycle. In this study, we used the 3CL^pro^ protein as a target to conduct high-throughput virtual screening of FDA-approved drugs to find potential drugs against COVID-19. According to the level of the docking score, 10 potential drugs that could inhibit SARS-COV-2 were identified. Among them, The Indinavir and 3CL^pro^ protein had more advantages in docking result and binding affinity. Studies by other people had also found that Indinavir and other FDA-approved drugs had potential advantages in the process of binding to 3CL^pro^ protein, which was consistent with our research, which indicated that drugs such as Indinavir could be used as the inhibitor of 3CL^pro^ protein in subsequent study [15, 16]. Unfortunately, their research lacked in-depth exploration of its binding mechanism, and the conformational differences of the 3CLpro protein after binding to Indinavir and other drugs were unclear.

Therefore, in order to explore the conformational differences of the 3CL^pro^ protein after binding with Indinavir, the 100 ns molecular dynamics simulations were performed on the 3CL^pro^ system and the 3CL^pro^-Indinavir complex system for the first time in this study, respectively, and the post-dynamic analyses of the simulated trajectories of the two systems was performed. Firstly, RMSD and RMSF analyses showed that both systems reached equilibrium around 5 ns, and the 3CL^pro^-Indinavir system had higher stability. Then, through PCA and DCCM, the conformational states of these two systems was explored, and it was found that the 3CL^pro^-Indinavir system had a smaller phase space and the negatively correlated movement of the system was significantly reduced. Finally, the RIN and H-bond occupancy were analyzed, and it was found that the residue-residue interactions in 3CL^pro^ protein increased significantly after binding with Indinavir, especially the catalytic dyads His41-Cys145 could form multiple stable H-bond and VDW interactions with surrounding residues., which revealed the deeper reason why Indinavir inhibited 3CL^pro^ protein. In short, although our research is based on bioinformatics, its effectiveness needs to be verified by subsequent *in vitro* tests. Through this research, we obtained some FDA-approved drugs that might have potential inhibitory effects on 3CL^pro^ protein, and further clarified the conformational differences of 3CL^pro^ protein after binding with Indinavir, which provided valuable information for subsequent drug development and optimization. We hope to provide more new clues against COVID-19.

## MATERIALS AND METHODS

### System preparation

The crystal structure of 3CL^pro^ protein (PDB ID: 6LU7) was downloaded from the Protein Data Bank (PDB) [9]. The protein was prepared through the " Prepare Protein " module in Discovery Studio (DS) v3.5 software, including removing water, adding the hydrogen atoms, assigning bond order, treating metals, treating disulfides, and inserting missing residues. In addition, ligands were prepared by DS v3.5 "prepare ligand" module, which included maintaining ionization, desalting, generating tautomer and converting the 2D conformation to the 3D conformation.

### High-throughput virtual screening based on molecular docking

Discovery Studio V3.5 software was used for high-throughput virtual screening. We downloaded all FDA-approved ligand structure models from the ZINC database. These compounds were prepared before docking screening.

The model of the interaction between protein and ligand was obtained by executing the "CDOCKER" module, which was based on the docking method of CHARMM. The "Define and Edit Binding Site" tool was used to define the binding pocket and select all amino acid residues within 5 Å around the ligand [17]. The key amino acid residues at the 3CL^pro^ protein binding site include His41, Met49, Tyr54, Phe140, Leu141, Asn142, Cys145, His163, Met165, Glu166, Leu167, Phe185, Asn187, and Gln192 [11]. The compounds were docked into the receptor protein and ranked according to the docking score, where the -CDOKER_ENERGY value indicated the docking score, and the higher the docking score, the better the compound bound to the protein.

### MD simulation

MD simulation was performed in the GROMOS96 43a1 force field using the GROMACS 4.5.5 software package [18]. Firstly, through the generated "topology" file, the non-bonding parameters (atom types and charges) and bonding parameters (bonds, angles, and dihedrals) were defined in the simulation. Then, all models were simulated in a closed dodecahedral box filled with explicit single-point charge (SPC) water molecules, with a minimum distance of 1 nm between the protein surface and the box boundary. To neutralize the system, the appropriate amount of counter ions was added to the system. Subsequently, the steepest descent method was used to minimize the energy of each system to 1000 kJ·mol^-1^/nm to ensure that there were no spatial collisions and inappropriate geometry inside the system. The position-restrained dynamics simulation (namely NVT and NPT) better balanced the solvent and ions around the protein model. Here, N was the number of particles, P was the system pressure, V was the volume, and T was the temperature. During the 100 ps NVT simulation, all systems were heated from 0 K to 300 K and stabilized at 300 K with the help of the thermostat. During the 100 ps NPT simulation, the constant pressure device was used to remove the constraints and keep the constant pressure at 1 bar. All bonds in the system were constrained by the LINCS algorithm [19]. Finally, 100 ns MD simulation was performed.

### Principal component analysis (PCA)

As a statistical method, PCA was widely used to reduce the dimensionality of data obtained from molecular dynamics simulations, and could be used to extract dominant modes in molecular motion [20, 21]. The first two eigenvectors (PC1 and PC2) of the maximum motions were obtained by projection to identify the motion of the protein. Each main movement was characterized by an eigenvector and an eigenvalue. The eigenvector meant the direction of motion, and the eigenvalue meant the contribution of a specific component to the overall motion of the complex [22, 23]. The ensemble formula used to generate the elements Cij with coordinates i and j as the covariance matrix was defined as follows:

*C*_ij_ = <(r_i_ − <r_i_>) (r_j_ − <r_j_>)>

Where, r_i_ meant the Cartesian coordinates of the ith Ca atom and r_j_ meant the Cartesian coordinates of the jth Cα atom; and the Angular brackets “<>” meant the time average of all relevant configurations during the entire simulation; and *i* and *j* meant the number of Cα atom. The dynamic motion of the atoms in the system was calculated from the simulation trajectories to analyze the conformational differences between the system during the simulation [24, 25]. This analysis was performed using Bio3D library [26].

### Dynamic cross-correlation map (DCCM)

Cross-correlation was a 3D matrix representation that could display time-related information between protein residues [27]. In biomolecules, the relative movement of Cα atoms helped to identify the binding area. DCCM was used to detect the time-dependent motions of all Cα atom pairs, and was calculated according to the following formula: [28, 29]

*C*_ij_ = <Δ*r*_i_ • Δ*r*_j_>/(<Δ*r*_i_^2^><Δ*r*_j_^2^>)^1/2^

In the formula, i and j indicated the i-th and j-th atoms, respectively. The Δi and Δj indicated the displacement vector corresponding to i-th and j-th atoms. And the <.......> indicated the ensemble average. The cross-correlation coefficient *C*_ij_ varied from -1 to 1 [30, 31]. A positive value of *C*_ij_ indicated that the motion of atoms was interrelated and in the same direction, and a negative value of C_ij_ indicated that the residues were inversely correlated [32].

### Residue interaction network (RIN)

The residue interaction network (RIN) was a reliable tool for identifying functional residues and analyzing changes in the structure of the interaction network between proteins and ligands [33, 34]. RIN analysis had been widely used to analyze a wide range of biological processes, including mutational effects, protein folding, intra protein domain-domain communication and catalytic activity [35]. RIN was visualized by Cytoscape software [36]. The average structure in the simulated trajectory was extracted to construct the RIN defined as the contact network. In the 2D diagram of RIN, nodes represent residues, and the lines between them represent the interactions between residues and residues, including H-bond interactions and VDW interactions [37].

### Binding free energy

The molecular mechanics Poisson Boltzmann surface area (MM-PBSA) method was used to calculate the binding free energy between residues and ligand [38]. The binding free energy could be calculated by the following formula:

∆G_bind_ = G_complex_ – G_receptor_ – G_ligand_

Here, G_complex_, G_receptor_ and G_ligand_ were the free energies of complex, receptor and ligand, respectively.

∆G = ∆E_gas_ + ∆G_sol_ − T∆S
∆E_gas_ = ∆E_int_ +∆E_ele_ + ∆E_vdw_
∆G_sol_ = ∆G_polar_ + ∆G_nonpolar_

Here, the binding free energy (∆G) contained gas phase free energy (ΔE_gas_), solvation free energy (∆G_sol_) and conformational entropy (T∆S). The free energy (∆E_gas_) consisted of three parts, including internal energy (∆E_int_), electrostatic interaction (∆E_ele_) and van der Waals force (∆E_vdw_). Solvation free energy (∆G_sol_) includes electrostatic solvation free energy (∆G_polar_) and nonpolar solvation free energy (∆G_nonpolar_) [23, 39].
